# Transcatheter closure of patent ductus arteriosus in preterm infants: results from a single-center cohort

**DOI:** 10.3389/fped.2023.1292623

**Published:** 2023-12-20

**Authors:** Marion Honnorat, Thomas Perouse-De-Montclos, Mohamed Bakloul, Camille Walton, Marine Butin, Philippe Vo Van

**Affiliations:** ^1^ Service de Réanimation Néonatale, HFME, Hospices Civils de Lyon, Bron, France; ^2^ Service de Cardiologie Pédiatrique, Hôpital Louis Pradel, Hospices Civils de Lyon, Bron, France; ^3^ Cardiologie pédiatrique, Hôpital Privé Natécia, Lyon, France; ^4^ INSERM U1111, CNRS UMR 5308, ENS de Lyon, Université Claude Bernard Lyon 1, Centre International de Recherche en Infectiologie, Equipe “Pathogénie des Infections à Staphylocoques”, Lyon, France

**Keywords:** neonate, cardiology, ductus arteriosus, transcatheter closure, surgical ligation

## Abstract

**Objective:**

To assess the success rate of patent ductus arteriosus (PDA) transcatheter closure in preterm infants and to describe the nature of procedural adverse events and short-term clinical status.

**Study design:**

All the preterm infants with PDA transcatheter closure were evaluated retrospectively between July 2019 and March 2023 in a single level III neonatal intensive care unit in France. The procedure was performed in the catheterization laboratory using venous canulation. We retrospectively collected data about the patients' characteristics, procedural outcomes and complications.

**Results:**

Twenty-five infants born between 23.4 and 32.0 weeks of gestational age (mean ± SD 26.3 ± 1.9 weeks) underwent transcatheter PDA closure. Their mean age and weight at the time of the procedure were 52 days (range 22–146 days) and 1,620 g (range 890–3,700 g), respectively. Successful closure was achieved in all but one patient. Procedure related complications were reported in 10 infants (40%), including 6 left pulmonary artery stenosis one of which required a balloon dilatation, two cardiac tamponades and two inferior vena cava thrombosis. Only two post-ligature syndromes occurred after the procedure. Two infants died one of which was related to the procedure.

**Conclusion:**

Transcatheter closure of a PDA is a valid alternative to surgical ligation due to its high success rate and low incidence of post-ligature syndrome. Nevertheless, we also report rare, although serious complications.

## Introduction

The ductus arteriosus constitutes a frequent and major issue in preterm infants. The patency of ductus arteriosus (PDA) exceeds 50% in infants born before 28 weeks of gestational age (GA) (1). Moreover, a hemodynamically significant PDA (hsPDA) conditions preterm infants to a cardiac failure state. It has been associated with neonatal morbidities such as pulmonary hemorrhage, intraventricular hemorrhage (IVH), necrotizing enterocolitis (NEC) and bronchopulmonary dysplasia (BPD) (1, 2). The mortality is higher in infants born before 29 weeks of GA with a hsPDA than those without (3). Consequently, the shared takeaway from the vast literature about PDA is that preterm infants would benefit from ductal closure. Despite numerous studies about indication, timing and modalities of ductal closure, management of PDA remains controversial without any consensus (4). Pharmacological treatment, including intravenous administration of cyclooxygenase inhibitors or acetaminophen, is effective in only 50%–70% of infants and efficiency decreases for those born before 32 weeks of GA. Besides, significant related side effects such as renal failure, gastrointestinal perforation and NEC might occur (5, 6). As for surgical ligation it is an established treatment but remains a significantly invasive procedure associated with critical complications such as post-ligature syndrome (7). Furthermore, it was shown to be an independent factor of BDP and poor neurodevelopmental outcomes (8–10). Conversely, active therapeutic abstention and conservative management might be considered as a valuable strategy (11).

In this context, transcatheter closure is a minimally invasive therapy that has emerged as novel (12, 13). It has become the procedure of choice among infants over 4 kg (12, 14). Recently, it has been applied safely and successfully in preterm infants, especially in extremely low-birth weight infants (13, 15–21). As such, we have implemented the procedure in our institution since July 2019 in replacement of surgical ligature.

The purpose of this study was to describe our clinical experience with transcatheter PDA closure and characterize the nature of related adverse events among our population of preterm infants.

## Materials and methods

A retrospective study was conducted in a single French level III neonatal intensive care unit (Hôpital Femme Mère Enfant, Hospices Civils de Lyon, Bron, France). Between July 2019 and March 2023, every infant who underwent a PDA transcatheter closure was included. Patients were proposed for a transcatheter closure if the PDA met at least E3 criteria of hemodynamic significance accordingly to McNamara echocardiographic score including: a transductal diameter over 1.5 mm, unrestrictive pulsatile transductal flow (ductal peak velocity <2.0 m/s), moderate left heart volume loading (left atrium: Aortic ratio >1.5) and absent or reversal diastolic flow in superior mesenteric artery (22). Additionally, they should have required a ventilation support, be subjected to growth restriction a kidney failure and/or a need for inotropic agents (1, 23).

Some patients in our cohort were also included in an ongoing French register, Premiclose 2. Clinical data were extracted from medical charts (ICCA software, Philips®, Suresne, France). Gender, GA and weight, at birth and at the time of procedure, type of ventilation and nutritional support, medications for PDA closure, need for inotropic agents, IVH, NEC, retinopathy and BDP were collected. Cranial echography protocol in our unit includes: four ultrasounds the first week, two the second week, one every week for one month and one every two weeks afterwards. The first dilated fundus examination is performed at 6 weeks of age. The follow up is then determined according to the result. Specifics about transcatheter closure including the Mcnamara score*,* type of device, ductal diameter in echocardiography and angiography, mean blood flow velocity in left pulmonary artery (LPA) and left atrium to aortic valve ratio were also gathered (22).

The primary outcome was the success rate defined as the closure of the PDA. Secondly, we described complications related to the transcatheter closure. Major complications were defined as procedural adverse events leading to death, organic failure, or long-term disabilities. Cardiac complications, as previously reported, were also collected (24). Cardiologists followed all patients immediately after the procedure and discharge. Finally, the third aspect was the evaluation of the short-term clinical status after the intervention: need for inhaled nitric oxide (iNO) or inotropic agents, kidney failure, weaning of respiratory support.

### Procedural technique 

Procedures were performed in the cardiac catheterization laboratory, by two experienced and senior cardiologists, under general anesthesia. Both anesthesiologist and neonatologist managed anesthesia, hemodynamic and ventilation support. Temperature was continuously monitored throughout the intervention. Cefazolin (30 mg/kg) was used for antibiotic therapy, and heparin 30–50 UI/kg was injected at mid-procedure. The vascular route was accessed via an anterograde echo-guided (Sonosite S and L25 mm 13–6 MHZ probe, Fujifilm, Netherlands) femoral venous puncture with a 4 F desilet. A 4 F vertebral catheter was used with a 0.018-inch Terumo guidewire (Terumo, Japan) to cross the PDA. The ductal diameter was measured by fluoroscopy. The amount of contrast injected was limited to preserve the renal function. The vertebral catheter was then exchanged for a 4 F Torqvue (Abbott, United States) delivery sheath through a 0.035-inch J guidewire.

Our center used a Amplatzer ADO II AS device, which was then named Amplatzer Piccolo Occluder by the manufacturer (Abbott, United States). Originally, Abbott's material sizing chart based on ductal diameter and length was used to select the device. An ADO II 4 mm waist diameter was chosen when the ductal diameter measured between 1.8 and 3.2 mm. Above 3.3 mm diameter, an ADO II 5 mm waist diameter was appointed. An ADO II 2 mm length was selected for DA shorter than 12 mm in length, and a ADO II 4 mm length for DA longer than 12.1 mm, respectively. Later, the cardiologists operate with shorter occluder than the ones recommended by Abbott's guide, based on their own experience. Yet the diameter of the disc remained determined accordingly to the manufacturer's sizing chart. The prothesis was deployed via echocardiographic guidance, which checked for any residual shunting and LPA or aortic obstruction before release. If so, the device was reloaded and repositioned.

### Ethics 

The Ethical Committee of the Hospices Civils de Lyon approved this observational study in March 2023 (number 22-5161). Written information about the study was sent to the patients's families, who were given the choice to decline the use of the patient's medical record. All data were anonymized.

### Statistical analysis 

Categorical data are reported as count and percentage. Continuous data are summarized by mean or median and SD, minimal and maximal values.

## Results  

### Demographic data

Between July 2019 and March 2023, 25 patients were included. Demographic details are shown in Table 1. The mean GA was 26 ± 1.9 weeks (range, 23.4–32 weeks). Most of the preterm infants weighted less than 1,000 g at birth (*n* = 22–88%). The mean age at procedure was 52 ± 30.8 days (range, 22–146 days) and the mean procedural weight was 1,620 ± 819.4 g (range, 890–3,700 g). Every high-grade cerebral lesion and retinopathy occurred before the procedure. BPD was diagnosed in 7 patients (28%) as well. Overall, 23 preterm infants (92%) developed BDP at the time of hospital discharge.

**Table 1:** Demographic data.

	Number or Mean	SD	Median	Min-Max
GA (weeks)	26.3	1.9	26	(23.4–32)
Birth weight (g)	747.7	162.8	700	(505–1,040)
Gender, female	13 (52%)			
PDA Medical treatment				
Ibuprofen	9 (36%)			
Acetominophen	6 (24%)			
Age at procedure				
Post-natal days	52	30.8	39	(22–146)
Postmenstrual age (weeks)	33.9	4.8	32	(28–46)
Procedural weight (g)	1,620	819.4	1,265	(890–3,700)
Inotropic agent	13 (52%)			
Exclusive Enteral Nutrition	17 (68%)			
NEC	6 (24%)			
BPD	7 (28%)			
Grade III and IV IVH	2 (8%)			
PVL	1 (4%)			
ROP	8 (32%)			

NEC, necrotizing enterocolitis; BDP, bronchopulmonary dysplasia; IVH, intraventricular hemorrhage; PVL, periventricular leukomalacia; ROP, retinopathy of prematurity.

Medical closure treatment was not systematically conducted before transcatheter procedure. Thus, ibuprofen (PEDEA, Recordati Rare Diseases, France) or acetaminophen (Paracetamol, B Braun, Germany) failed to close PDA in 15 patients. Six of them were contra-indicated for Ibuprofen due to renal, neurological or intestinal causes. Hence, Acetaminophen was the treatment of choice.

Mean echocardiographic PDA diameter was 2.7 mm at the pulmonary end. PDA characteristics are shown in Table 2. Every patient was scored according to the clinical and echocardiographic staging system adapted from McNamara and al (22). All PDA were considered as hemodynamically significant (graded E4). In most cases, they were symptomatic (graded C3), mainly responsible for ventilation weaning difficulties. Only 3 patients displayed a mild clinical PDA and one was clinically asymptomatic. Finally, three others had a bidirectional ductal flow (patient number 2, 12 and 24). Catheter closure was also indicated for respiratory reasons.

**Table 2:** PDA characteristics.

Characteristics	
PDA Diameter (mm)	Echocardiography 2.7 (2–3,7)
LA: Ao ratio	1.8 (1.1–2.3)
LPA mean velocity (m/s)	0.7 (0.37–1.1)
Left to right flow	22 (88%)
Bidirectional	3 (12%)
Clinical staging system (McNamara and Hellman): C1, C2, C3, C4	3
Echocardiography staging system (McNamara and Hellman): E1, E2, E3, E4	4

### Catheter procedure outcomes

Implant success rate was 96% (24 infants, see Supplementary Table). We reported adverse effects in 40% of all patients (10 infants) with a various range of severity. Mean age and weight were 39 days and 1,210 g respectively at the time of the procedure for neonates with complications. On the one hand, 6 cases were minor and transient with no clinical consequences. On the other hand 4 major complications occurred in 3 infants. One case of extended venous thrombosis and 2 cases of severe cardiac failures are described below. Mortality rate was 8% (2 infants), only one was related to the procedure. The other one died from a sudden infant death syndrome at 6 months old.

The most frequent complication was LPA stenosis, in 24% (6 infants). Mean procedural weight for neonates with LPA stenosis was 1,150 g. They all occurred right after procedure, due to protrusion of the device in the LPA. Two of them had mild LPA flow acceleration (1.5–2 m/s), 3 had stenosis (>2 m/s). Follow-up echocardiography was performed at 1 week, 1 month and then every 2 months for infants with LPA stenosis. Four had a favorable outcome. One infant had a significant LPA stenosis with an asymmetrical pulmonary perfusion, requiring a balloon dilatation at one year old. At four years old a minor asymptomatic stenosis remained. Such complications happened between 2019 and 2021 and eventually disappeared over time except for one patient described below.

The minor adverse events consisted of mild hemolysis and a left ventricular dysfunction without any need for inotropic agents immediately after the procedure. No related infections were reported. Finally, one case of progressive arterial hypertension started four days after closure, in a 26 GA preterm infant with a history of omphalocele and unilateral kidney agenesis. Amlodipine was administered for one month with a favorable outcome.

#### Case number 6

Infant number 6 was born at 25 GA, weighing 795 g. Ibuprofen prescribed at four days old failed to close the PDA. A 2.2-mm hsPDA with high pulmonary blood flow was then diagnosed by follow-up echocardiography. Despite diuretics and hydric restriction, transcatheter closure was decided due to ventilatory difficulties at 28.5 weeks PMA (24 days old) and 1,025 g. The closure was performed successfully but within 24 h after the procedure a macroscopic hematuria and acute renal failure occurred. Maximal creatinine was increased up to 0.331 mmol/L. Inferior vena cava (IVC) thrombosis associated with bilateral renal vein thrombosis were diagnosed by doppler ultrasound. The outcome was favorable after 3 months of heparin treatment. Eventually, the renal function normalized despite a left renal atrophy.

#### Case number 24

Infant number 24 was born at 23.5 GA, weighing 570 g. He remained on mechanical ventilation and suffered from an extra-uterine growth retardation. Similarly, he was diagnosed with a 2.6 mm hsPDA. Diuretics and hydric restriction managed to partially limit the high pulmonary blood flow and the left heart pressure overload. However, ductal catheter closure was decided due to the lack of clinical progress. The procedure was performed at 29 weeks PMA (38 days old) and 1,045 g. The infant was relatively unstable, needing a high frequency oscillation ventilation and norepinephrine. The procedure was complicated by severe LPA stenosis and traumatic tricuspid regurgitation. Retrieval of the occluder failed and was rapidly postponed due to the clinical instability of the patient. Additionally he developed transient post-PDA ligation syndrome during the first 24 h. A second transcatheter attempt to retrieve the occluder was scheduled 48 h later. It proved unsuccessful as well. Moreover, a lesion through the right ventricle infundibulum caused a tamponade leading to an emergency drainage surgery and ductal ligation. The patient died shortly after.

#### Case number 25

Infant number 25 was born at 27.2 GA, weighing 970 g. He was a candidate for a transcatheter closure due to a 3.2-mm hsPDA. In addition, he needed assisted ventilation for repeated bradycardia and its growth's charts showed stagnation. The catheter procedure was scheduled at 31.6 weeks PMA (30 days old) and 1,265 g. PDA measured 5.1 mm in fluoroscopy. The device was positioned in the DA but did not sufficiently obstruct it, thus preventing its release. This caused a risk of systemic embolization. During the final attempt, the guidewire and the delivery sheath swapped their initial trajectory and got stuck into the right ventricle infundibulum causing a tamponade. The pericardial effusion was limited as long as the sheath stayed into the infundibulum. An emergency sternotomy to drain the pericardial effusion and ligate the DA was performed. The patient survived. Inotropic support was needed for 48 h. He also developed an IVC thrombosis diagnosed by a systematic doppler ultrasound. Heparin treatment was conducted leading to a favorable outcome. The only symptom was an isolated macroscopic hematuria.

### Pre- and post-transcatheter clinical status

Pre-closure status and clinical status 24 h post-closure are shown in Table 3. Thirty two percent (8 infants) were on mechanical ventilation 24 h prior PDA closure: two on conventional ventilation and six on high frequency oscillation. All transcatheter procedures were performed on assisted ventilation. The mean delay of extubation was 3 days. The two patients who suffered from post-ligation syndrome and needed inotropic agents are described above. One of them had ductal surgery. Twenty percent (5 infants) endured pulmonary hypertension treated by iNO after procedure. One of them had mild LPA stenosis and required iNO for 3 days. Two didn't encounter any procedural complications and iNO was discontinued after 10 days. Finally, the last 2 patients were number 24 and number 25 and are described above.

**Table 3:** Clinical status.

	Pre closure status (24 h)	Post closure status (24 h)
Type of Ventilation		
Non invasive	15 (60%)	1 (4%)
Mechanical ventilation	9 (36%)	24 (96%)
FiO2 (%)	60%	47%
iNO	0 (0%)	5 (20%)
Creatinine (mmol/L)	0.05 **±** 0.03 (0.014–0.146)	0.05 **±** 0.03 (0.015–0.142)
Inotropic agent	1 (4%)	2 (8%)
Duration of mechanical ventilation post procedure (Days)		3 **±** 3.45 (0.08–13)

iNO, inhaled nitric oxide.

Data presented as mean ± SD, range [minimum, maximum] for continuous variables or number of patients (percentage).

## Discussion  

This study reports on a sample of preterm infants who underwent a transcatheter ductal closure in a French level III neonatal intensive care unit. It documents a high rate of successful technical outcome but also a few major complications. Thus it brings new insights on the recent percutaneous ductal procedure in neonates.

The previously adopted strategy for ductal management usually associated early screening with conservative management (11, 25). HsPDA were treated if they were symptomatic within the first days of life. Moreover, surgical ligation was avoided in most of the patients as the ductal shunt tended to close spontaneously. Nevertheless, it is most likely that PDA played a role in the mortality and the population's BPD. Therefore, the transcatheter closure procedure has been progressively implemented since July 2019 as an alternative to surgery to avert the post ligature syndrome (7, 26).

The reported successful rate is 96%. It correlates with previous studies and therefore confirms the feasibility of transcatheter closure in preterm infants (15, 27, 28, 29). Neither the weight nor the GA constitute a technical impediment to the procedure. Also, feasibility of transcatheter ductal closure swiftly increased with the occluders' miniaturization (17, 18, 29, 30). Indeed, it has emerged as a standard procedure in the United States (13, 26, 29, 31).

The mean age at the time of catheterization was 52 days old with a width standard deviation of +/−30.8 days. This age data vary between studies. On the one hand, numerous studies have a mean procedural age between 19 and 39 days (16, 27, 32, 33). Sathanandam et al. suggested that closure applied earlier than 4 weeks old may benefit infants capable of a quicker recovery by avoiding increased pulmonary artery pressure (33). Furthermore, Regan et al. showed that patients with an early ductal closure were discharged home before those who underwent closure later in life (34). On the other hand, our results are similar to those which defer the intervention after the first month (17, 28). Our center had been managing PDA with a conservative approach involving fluid restriction and diuretics. Besides, a non-intervention strategy has been deemed a reasonable alternative to mandatory closure, particularly due to the relatively high rate of spontaneous ductal closure (35–38). Last, we had assimilated both transcatheter closure and surgery as invasive procedures. Since surgery has been associated with BPD and post ligature syndrome that decreased after 4 weeks old, the same behavior was assumed to apply to transcatheter procedures (9, 39–41). Therefore, percutaneous procedure would be postponed after 4 weeks*.* Yet such an assumption appeared to be false. It is worth noting that only two patients (8%) needed inotropic agents after the intervention. One of them had already been administered inotropic agents prior. The other one had undergone a surgical ligation after a failed transcatheter closure complicated by a tamponade. This confirms previously published data which showed that post ligature syndrome occurred less frequently after percutaneous closure compared to surgery (28, 42–44). Furthermore, the delay time of extubation was rather short with a mean of 3 days. Similarly, Rodriguez and al. demonstrated that patients who underwent a catheter procedure achieved a faster pulmonary recovery than those who were operated on (28). The latter also required more respiratory support than before the surgery. Post-ligature syndrome is related to a left ventricular dysfunction (7). The acute and sudden interruption of the ductal left to right shunt increases the left ventricular afterload, which impairs both systolic and diastolic function. The systolic dysfunction reduces the left ventricular output whereas the diastolic impairment raises the pulmonary venous pressure and leads to pulmonary oedema. We hypothesize that percutaneous procedure allows a more progressive ductal closure as residual shunts may persist along with post-interventional echocardiographic control. Thus, it enables the heart to adapt to the new hemodynamic balance. Serrano and al. also speculated that a percutaneous procedure avoids the manipulation of the lung secondary to a thoracotomy. Hence it decreases the subsequent inflammation and the associated cardiopulmonary stress (43).

Procedure-related complication rate was high in our cohort (40%). However, the majority (60%) was minor without any clinical consequences. They also seem to occur more likely in smaller infants as their mean weight at the time of procedure were below the rest of our population (1,210 g vs. 1,620 g). However, the small number of infants in our cohort unable us to draw any statistical conclusions. Severe complications occurred in 3 patients. Cardiac tamponades have been described in previous studies (24). They remain rare but are associated with a high mortality rate. This complication occurred in 2023 and hence was not related to the cardiologists' lack of experience. After consideration, they switched the 0.018-inch Terumo guidewire to a less rigid 0.014-inch Sion Blue coronary guidewire (Asahi Sion Blue, Japan). Finally, the early intervention of the cardiac surgeons was a major asset especially for the survival of one of our patients. Thus, we recommend performing transcatheter PDA closure in a cardiac surgery center. Two infants also developed IVC thrombosis, which evolved favorably with heparin. In infant number 6 we concluded that the desilet was inserted too far up to the right atrium, which had potentiated the thrombosis. Therefore, the desilet size was changed enabling a reduction of venous thrombosis in our cohort. Kim et al, described an infrarenal IVC thrombosis and a retroperitoneal hematoma occurring while advancing the sheath. The patient required multiple blood transfusions and inotropic support (42). However, no heparin treatment was mentioned. Regan and al. also reported one patient with a left femoral vein thrombosis, which spontaneously resolved without any treatment (34). Additionally, from the experience of infant number 6, we decided to systematically perform a venous doppler ultrasound within 48 h of the procedure completion. This practice allowed to detect the IVC thrombosis in infant number 25. Such routine monitoring of venous doppler ultrasound was also proposed by Sathanandam et al. but was ultimately abandoned due to lack of thrombosis events (33).

LPA stenosis occurred more frequently (24%) than in other studies (2%–10%) (15, 17, 18, 21, 30). However, most of them were clinically insignificant and resolved over time thanks to adequate growth of the vessel. LPA obstruction constitutes the most frequent complication due to the protrusion of the occluder. In low-weight infants LPA often measures less than the retention disc (29). As the matter of fact, patients with LPA stenosis weighted less than the cohort's average (1,150 g vs. 1,620 g). In a Taiwanese cohort, younger age and smaller body weight at the time of procedure were also associated with a risk of LPA stenosis (21). Besides a large DA restricted at the pulmonary end potentiates a protrusive mechanism (20). On the other hand, no aortic coarctation was reported in this study unlike the others (16, 34, 45). The risk of LPA obstruction is counter-balanced by the risk embolization and aortic coarctation which makes the choice of the occluder's diameter critical. Furthermore, during the first procedures, the choice of the occluder was based on Abbott's guide accordingly to the ductal diameter and length. It allowed to determine the right diameter of the discs and length of the prothesis. However, while analyzing the occurrence of LPA stenosis, the cardiologists found that the size of the occluder suggested by the manufacturer might have been too long. Abbott recommends a 5/4 ADO Piccolo in DA longer than 12 mm. As a consequence, the first disc could have procluded onto the LPA. On the contrary a shorter prothesis would enable the deployment of the disc directly into the DA. Hereafter, the cardiologists chose a shorter prothesis [ADO Piccolo (5/2)] in length than the one determined by Abbott's sizing chart since patient 7. Ado Piccolo 5/4 occluders were reserved for DA above 20 mm. Nevertheless, they still followed the manufacturer's recommendation regarding the diameter. Since patient 7 in 2021, no other LPA stenosis except for one (case number 24) occured. Finally, echocardiography plays a major role in identifying those complications and confirming the successful position of the implant.

This study has the inherent limitations of a retrospective analysis conducted with a limited sample size in a single institution. Furthermore, it analyzes a brief experience with transcatheter closure, which may result in an overestimated complication rate. Nonetheless, it shows that transcatheter closure can be successfully performed in low-weight preterm infants. Before the availability of this procedure, only a few patients in our center underwent surgical ligation. It was reserved for the most severe cases, due to the complications encountered in post-operative management and the rate of spontaneous PDA closure. In this regards, interventional catheterization offers an interesting alternative in the PDA treatment strategy. Further studies are needed to define clinical and hemodynamic criteria of closure as well as its adequate timing (4). They should also evaluate the benefits on neonatal comorbidities such as BPD.

## Data Availability

The original contributions presented in the study are included in the article/**Supplementary Material**, further inquiries can be directed to the corresponding author.
