# Supplementary material for: Transcatheter closure of patent ductus arteriosus in preterm infants: results from a single-center cohort
Source: Front Pediatr. 2023 Dec 20;11:1292623. doi: 10.3389/fped.2023.1292623 (PMC10765538; doi:10.3389/fped.2023.1292623)
Supplement: Supplementary file 1 [file Table1.docx]

Supplementary Table. Catheterization data and outcome. BPD: bronchopulmonary dysplasia, iNO : inhaled nitric oxide IVC: inferior vena cava, LPA: left pulmonary artery, TTE: transthoracic echocardiogram.

| **Patient number** | **Gestational age, weeks** | **Birth weight, grams** | **Age at procedure, days** | **Weight at procedure, grams** | **PDA length (Angiography), mm** | **PDA minimal diameter (TTE/Angiography), mm** | **Flow across the ductus** | **Device** | **Outcomes** |  |
| --- | --- | --- | --- | --- | --- | --- | --- | --- | --- | --- |
| 1 | 28.6 | 595 | 57 | 1590 | 10 | 2.5/4.5 | Left-to-right | ADO II AS (5/4) | LPA stenosis, BPD |  |
| 2 | 24.5 | 505 | 52 | 1100 | 13 | 2/3.1 | Bidirectional | ADO II AS (5/4) | LPA stenosis, BPD |  |
| 3 | 29 | 1040 | 54 | 2080 | 11 | 2.8/3.5 | Left-to-right | ADO II AS (5/4) | Favorable, BPD |  |
| 4 | 25.5 | 690 | 39 | 1030 | 9 | 2.8/2.9 | Left-to-right | ADO II AS (4/2) | Favorable, BPD |  |
| 5 | 26.1 | 850 | 37 | 1500 | 10 | 2.8/2 | Left-to-right | ADO Piccolo (4/2) | Favorable |  |
| 6 | 25 | 795 | 24 | 1025 | 13 | 2.2/3 | Left-to-right | ADO Piccolo (4/2) | IVC thrombosis, acute renal failure, BPD |  |
| 7 | 28.4 | 1010 | 25 | 1590 | 16 | 3/3.4 | Left-to-right | ADO Piccolo (5/2) | Favorable, BPD |  |
| 8 | 27.2 | 700 | 36 | 1000 | 14 | 3/4.3 | Left-to-right | ADO Piccolo (5/2) | LPA stenosis, mild hemolysis, BPD |  |
| 9 | 32 | 840 | 43 | 1400 | 12 | 2.8/3.4 | Left-to-right | ADO Piccolo (5/2) | Favorable |  |
| 10 | 24.6 | 660 | 22 | 890 | 14 | 2.5/4 | Left-to-right | ADO Piccolo (5/2) | Favorable, BPD |  |
| 11 | 24.4 | 745 | 146 | 3700 | 21 | 2.9/3.6 | Left-to-right | ADO Piccolo (5/4) | Favorable, BPD |  |
| 12 | 24 | 625 | 119 | 2035 | 19 | 2.5/3.9 | Bidirectional | ADO Piccolo (5/2) | Favorable, BPD |  |
| 13 | 26 | 680 | 38 | 1000 | 9 | 2.6/2.5 | Left-to-right | ADO Piccolo (3/2) | Favorable, BPD |  |
| 14 | 26 | 505 | 38 | 955 | 6 | 2/3 | Left-to-right | ADO Piccolo (4/2) | transient pulmonary hypertension iNO, BPD |  |
| 15 | 24.6 | 640 | 47 | 1280 | 12 | 2.5/3.5 | Left-to-right | ADO Piccolo (5/2) | LPA stenosis, BPD |  |
| 16 | 25.3 | 590 | 27 | 895 | 17 | 2.3/3.9 | Left-to-right | ADO Piccolo (5/2) | LPA stenosis, transient pulmonary hypertension iNO, BPD |  |
| 17 | 27.5 | 955 | 85 | 3210 | 23 | 2.7/4.1 | Left-to-right | ADO Piccolo (5/4) | Favorable, BPD |  |
| 18 | 26.2 | 750 | 34 | 1200 | 9.5 | 2.4/4.4 | Left-to-right | ADO Piccolo (5/2) | Favorable, BPD |  |
| 19 | 26 | 900 | 38 | 1200 | 11 | 3/3.2 | Left-to-right | ADO Piccolo (4/2) | Mild left ventricular dysfunction, BPD |  |
| 20 | 26.6 | 650 | 41 | 1700 | 20 | 3.7/5 | Left-to-right | ADO Piccolo (5/2) | Progressive arterial hypertension, BPD |  |
| 21 | 28 | 1000 | 95 | 3300 | 14 | 2.2/3.6 | Left-to-right | ADO Piccolo (5/2) | Favorable, BPD |  |
| 22 | 28 | 880 | 95 | 3100 | 18 | 3.3/4 | Left-to-right | ADO Piccolo (5/2) | Favorable, BPD |  |
| 23 | 23.6 | 550 | 42 | 1070 | 9.8 | 2.1/3 | Left-to-right | ADO Piccolo (4/2) | Transient pulmonary hypertension iNO, BPD |  |
| 24 | 23.4 | 570 | 38 | 1045 | 13 | 2.7/3.7 | Bidirectional | ADO Piccolo (5/2) | Traumatic tricuspid regurgitation, severe LPA stenosis, pulmonary hypertension iNO death |  |
| 25 | 27.2 | 970 | 30 | 1265 | 18 | 3.5/5.1 | Left-to-right | none | Tamponade, surgical ligation, IVC thrombosis, pulmonary hypertension iNO, BPD |  |
